# Clinical review: peripheral muscular ultrasound in the ICU

**DOI:** 10.1186/s13613-019-0531-x

**Published:** 2019-05-17

**Authors:** Paolo Formenti, Michele Umbrello, Silvia Coppola, Sara Froio, Davide Chiumello

**Affiliations:** 1 SC Anestesia e Rianimazione, Ospedale San Paolo – Polo Universitario, ASST Santi Paolo e Carlo, Milan, Italy; 2 Dipartimento di Scienze della Salute, Università degli Studi di Milano, Milan, Italy; 3 Centro Ricerca Coordinata di Insufficienza Respiratoria, Università degli Studi di Milano, Milan, Italy

**Keywords:** ICU-acquired weakness, Skeletal muscle, Peripheral muscular ultrasound, Muscle cross-sectional area, Muscle layer thickness, Pennation angle, Muscle echointensity

## Abstract

Muscular weakness developing from critical illness neuropathy, myopathy and muscle atrophy has been characterized as intensive care unit-acquired weakness (ICUAW). This entity occurs commonly during and after critical care stay. Various causal factors for functional incapacity have been proposed. Among these, individual patient characteristics (such as age, comorbidities and nutritional status), acting in association with sustained bed rest and pharmacological interventions (included the metabolic support approach), seem influential in reducing muscular mass. Long-term outcomes in heterogeneous ICUAW populations include transient disability in 30% of patients and persistent disabilities that may occur even in patients with nearly complete functional recovery. Currently available tools for the assessment of skeletal muscle mass are imprecise and difficult to perform in the ICU setting. A valid alternative to these imaging modalities is muscular ultrasonography, which allows visualization and classification of muscle characteristics by cross-sectional area, muscle layer thickness, echointensity by grayscale and the pennation angle). The aim of this narrative review is to describe the current literature addressing muscular ultrasound for the detection of muscle weakness and its potential impact on treatment and prognosis of critically ill patients when combined with biomarkers of muscle catabolism/anabolism and bioenergetic state. In addition, we suggest a practical flowchart for establishing an early diagnosis.

## Background

Many patients admitted to the intensive care unit (ICU) develop a syndrome of neuromuscular dysfunction characterized by generalized muscle weakness that often contributes to difficult liberation from mechanical ventilation [1]. The principal causes of such weakness include neuropathic and myopathic disorders, as well as mixed disorders that have been lumped under the term “ICU-acquired weakness” (ICUAW) [2]. Since this syndrome occurs in the absence of pre-existing neuromuscular disease and affects all age groups [3–6], ICUAW is believed to reflect illnesses or treatments occurring in the ICU [7]. Recent studies demonstrated that a reduced excitability of the nerve and muscle cell membranes might contribute to the development of ICUAW during the acute stages of the polyneuropathy and myopathy encountered in critically ill patients [8–11]. This association has been linked with severe systemic responses to infection, as well as with mortality rates of 30–50% and transient disability in 30% of the cases [12]. Persisting disabilities often linger in patients with apparently complete functional recovery [13]. Moreover, ICUAW is now recognized to be a very important factor in “difficult-to-wean” patients in the ICU setting, associated with prolonged ICU/hospital stays [14, 15]. Regarding the development of ICUAW, many factors play a key role, even if the pathogenesis of this syndrome is far from being completely understood [2, 16]. Among these, individual patient features, such as age and comorbidities [17, 18], sustained bed rest [19], pharmacological strategies, nutritional and metabolic support, all seem to be related with the reduction in muscle mass [20]. In order to improve nutritional and rehabilitation strategies, different diagnostic methods to diagnose ICUAW at an early stage have been investigated [21–23]. The currently available tools for the assessment of skeletal muscle mass with the highest level of accuracy and reproducibility are computerized tomography (CT) and magnetic resonance imaging (MRI); however, high-precision imaging is difficult to perform in the ICU setting. On the contrary, high-resolution ultrasound machines that are now routinely available represent valid and reliable tools for providing qualitative and quantitative details about muscle disease [24–28]. Few systematic reviews have been focused on this topic [29, 30], and the authors did not perform formal meta-analyses due to issues with the design of the studies included, the heterogeneity of patient cohorts, and the different aspects of peripheral skeletal muscle architecture measured. Since then, few papers have focused on the validation of methodologies [28, 31] and others investigated different muscle zones and ultrasound characteristics [32–35]. Ultrasound is able to derive structural variables, and, once it is performed repeatedly, it is able to identify those patients at highest risk of prolonged complications, which result from excess muscle catabolism. Thus, the aim of this narrative review is to describe the current literature about the use of muscular ultrasound for detecting muscle weakness and its impact on critically ill patients’ treatments and prognosis. Moreover, we propose a flowchart with the aim of using muscular ultrasound together with clinical examination for an early diagnosis of pathology and etiology.

## Ultrasound assessment of muscular features

**Fig. 1:**
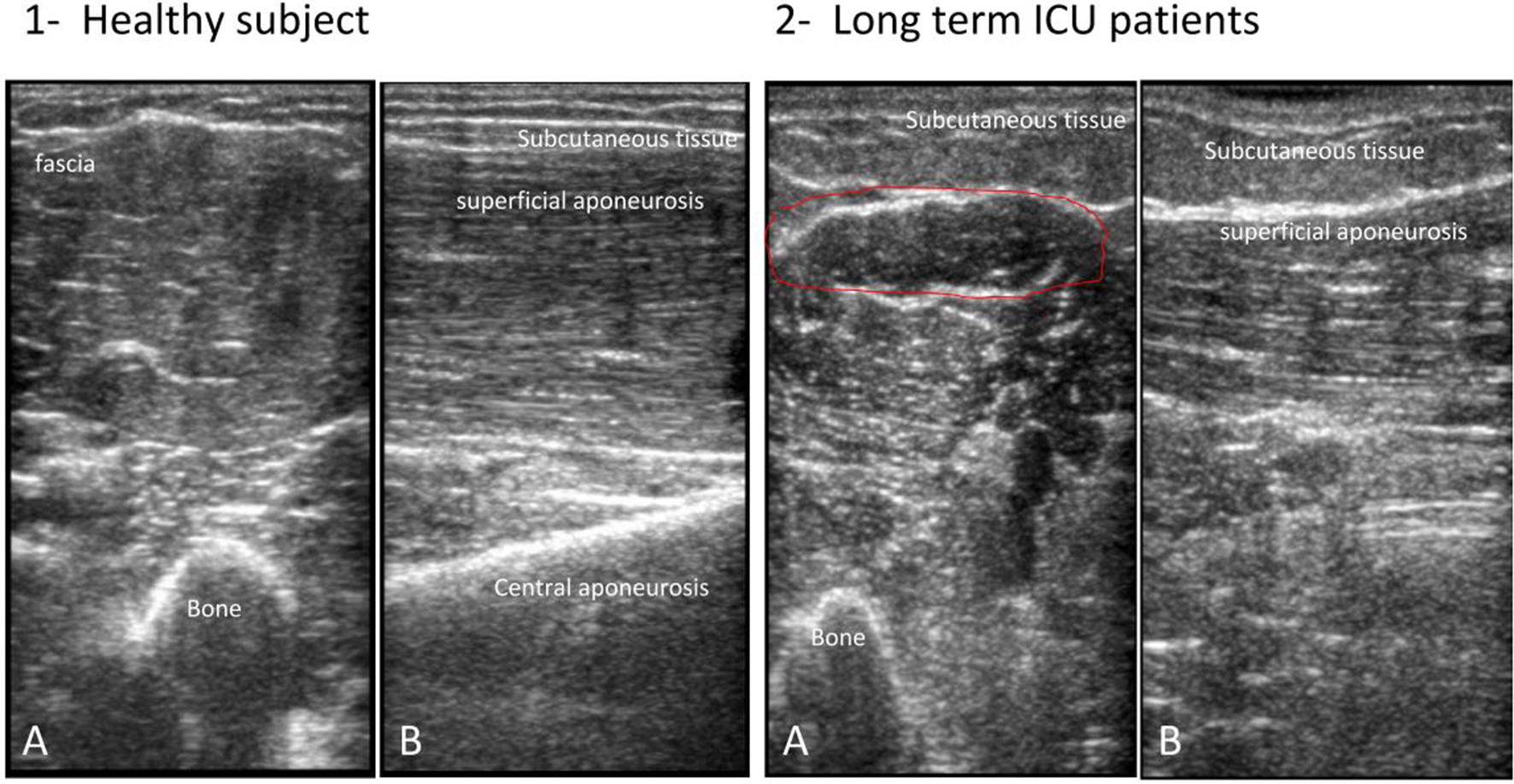
Ultrasound distinctive appearance of muscle tissue. The figure shows a transverse (**a**) and longitudinal (**b**) ultrasound scan of elbow flexor (bicep brachialis) in healthy (1) and long-term ICU (2) subjects. In the axial image, muscle consists of primarily hyper-echogenic areas scattered with small bright curved echoes of superficially random orientations. In the sagittal plane, these bright echoes are seen to be the fibrous tissue that surrounds muscle fibers and fascicles and which organize into recognizable striations. In bipennate or multi-pennate muscles, a central aponeurosis can be identified as an area of thickened fibrous tissue that when followed distally becomes the tendon. Bone is highly echogenic with a deep shadow beneath the bright hard edge. Subcutaneous fat is typically of similar echogenicity to muscle and is interposed with brighter, poorly organized strips of connective tissue. Near the myotendinous junction, the myofascial fibrils merge, resulting in increased echogenicity and higher anisotropy. In the healthy tissue, the hyper-echogenic muscle is interspersed with bright fibro adipose tissue and the bone reflection is bright and sharply defined; in the long-term ICU patient, the muscle tissue appears as non-homogenous and reduced in its mass

As a noninvasive, painless technique, ultrasound may be used to identify skeletal muscle pathology. It offers several advantages compared with other tests used in the evaluation of muscle features and allows for a quick screen of large muscle areas at the bedside. In fact, healthy muscle tissue has a distinctive appearance on ultrasound that readily distinguishes it from other tissues (Fig. 1) [36, 37]. To perform an adequate ultrasound examination of skeletal muscle, several technical components must be considered. First, since muscle and subcutaneous fat can easily be compressed, a minimal amount of pressure should be applied on the tissue under an ultrasound probe sufficiently covered with gel, in order to optimize imaging conditions. Additionally, obesity and subcutaneous edema can significantly alter the appearance and quality of the ultrasound images of skeletal muscle. Therefore, the examiner must be aware of the depth of the imaged tissue, the potential effects of attenuation of the ultrasound signal, and the limitations of the ultrasound system in use, taking into account the gain, the focal points and the compression, as all of these factors may significantly alter the overall appearance of myofascial structures [24]. The probe orientation and muscle position can also radically alter the image appearance, given that the relationship between the probe angle and the underlying pennation angle of the myofascial strips critically modulates the ultrasonographic brightness of the muscle [38]. The most important factors that contribute to the heterogeneity in muscle detection are the transducer selection frequency and the field of view. Using a linear probe, the frequencies for the clinical evaluation of neuromuscular measures range from approximately 2–20 MHz. Radiologic imaging is increasingly used as a diagnostic tool to describe the patterns and extent of muscle involvement, thanks to modern techniques that enable determinations of muscle atrophy and changes in connective tissue. Such methodologies usually grade the severity of the disease process with greater accuracy than clinical scores. CT has made possible the evaluation and selective involvement of muscle groups, establishing disease progression and identifying asymptomatic involvement [39]. CT scan is usually more accessible than MRI. Moreover, it allows for quick and accurate evaluation of muscle changes, especially fatty degeneration, by assessing muscle density and morphology. Such examinations are not operator dependent and allow for the evaluation of the deepest muscles. However, CT scanning involves high doses of ionizing radiation. MRI provides excellent soft tissue characterization, so it perfectly evaluates shape, volume and morphological features of normal skeletal muscle allowing for determination of fatty degeneration in late-stage muscle dystrophy, aside from recognition of decreases in muscle volume [40]. Unfortunately, MRI incurs high cost and lacks portability. Cumbersome study logistics precludes its routine clinical use in the ICU setting. Nerve and muscle biopsies are invasive and expensive, hold potential for complications, and require specialized expertise for obtaining and interpreting samples. Although the classical diagnostic tool to detect ICUAW is the combination of nerve conduction and electromyographic studies (EMG), there are many technical challenges to completing both in the ICU [41]. Commonly encountered physiologic changes in the ICU, such as anasarca and hypothermia, introduce artifacts by altering amplitude and velocity recordings for nerve conduction studies [42]. Finally, the utility of manual muscle strength assessment using standardized scales such as that of the Medical Research Council proves useful in patients who are both sufficiently awake and cooperative [43]. A recent study investigated the accuracy of muscular and nerve ultrasound for the diagnosis of ICUAW compared with this benchmark test [44]. That study showed the MRC to have very low diagnostic accuracy for all muscle and nerve parameters. Considering the limitations of traditional methods, together with recent advances in muscular ultrasound, makes ultrasonography a promising tool for the study of muscle structure, facilitating early diagnosis and intervention.

## Parameters of muscle architecture

### Cross-sectional area

**Fig. 2:**
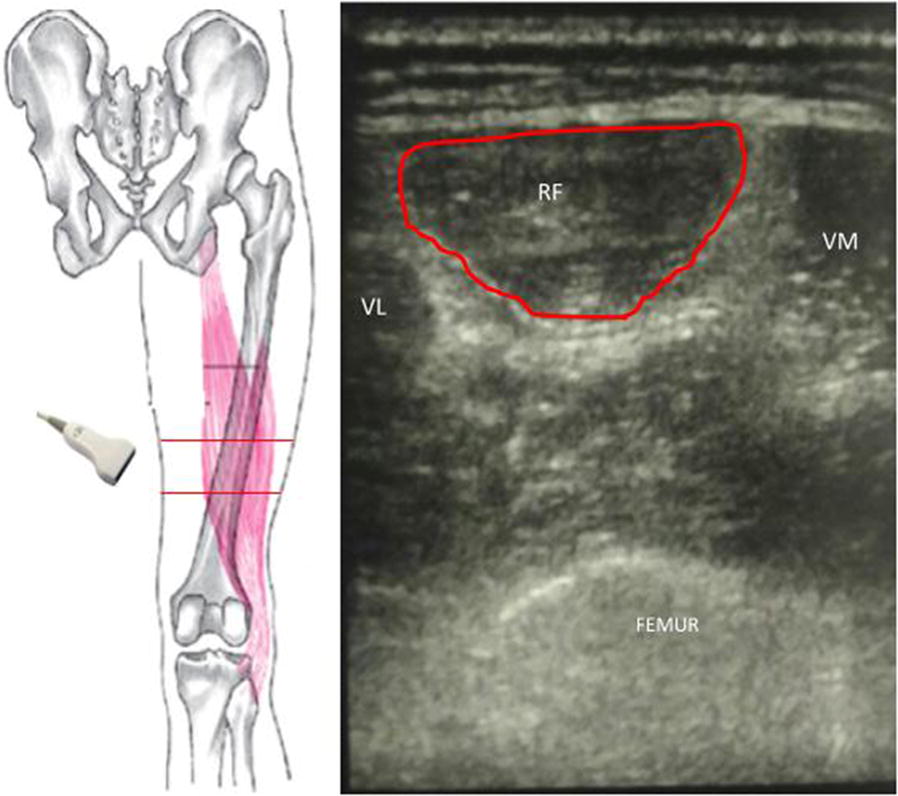
Muscle cross-sectional area. This figure depicts the cross-sectional area of the rectus femoris perpendicular to its longitudinal axis. The quadriceps femoris is a group of muscles composed by three vastus muscles (medialis, intermedius, and lateralis) and the rectus femoris which presents a proximal insertion in the anterior inferior iliac spine and other insertion in the supra-acetabular sulcus. Left side: standardized level of ultrasound scan of the lower limb; in the supine position, the probe should be placed at 2/5 of an imaginary line between the anterior parts of the thigh from the anterior inferior iliac spine to the midpoint of the proximal border of the patella. Right side: the figure depicts the cross-sectional area (red circle) of the rectus femoris (RF) perpendicular to its longitudinal axis. *VI* vastus intermedius, *VM* vastus medialis, *VL* vastus lateralis

The cross-sectional area (CSA) is determined by the number and size of individual fibers within a muscle. It is comprised of two areas: anatomical (cross section of a muscle perpendicular to its longitudinal axis) and physiological (cross section of a muscle perpendicular to its fibers, generally at its largest diameter). The term ‘muscle architecture’ (parallel or pennate) refers to the physical arrangement of muscle fibers at the macroscopic level and determines the muscle’s mechanical function. In a parallel muscle, the two CSAs coincide, as the fibers are parallel to the longitudinal axis. In pennate muscles, both areas may be used to describe the contraction properties (Fig. 2). In fact, since muscle strength relates to muscle volume, the latter may be inferred from its CSA [30]. Because these measurements do not need muscle tension, they are often assessed instead of muscle strength tests [45], especially in non-cooperative patients. Muscle atrophy mainly affects fast fibers (type II) rather than a relatively equal loss of slow and fast fibers. This loss potentially results in a drop in physical activity levels, in the denervation/re-innervation process, and in reduced synthetic rates of muscle proteins. Thus, muscle bulk can be measured by the CSA, whose variation is dependent on age, gender, and muscle group [46].

### Muscle layer thickness

**Fig. 3:**
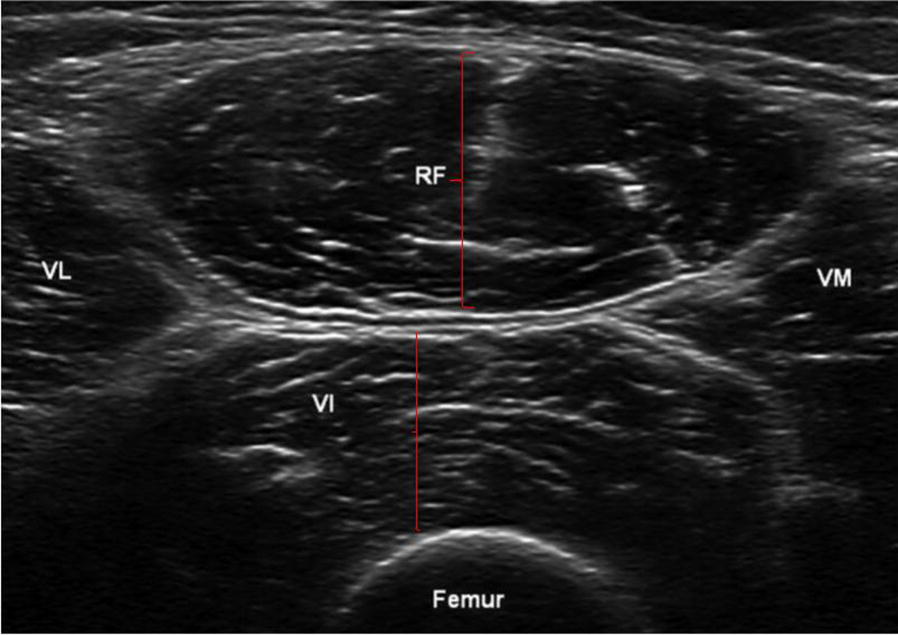
The muscle layer thickness detected by ultrasound. Quadriceps femoris detected by ultrasound in a transverse scan. The rectus femoris (RF) layer thickness and vastus intermedius (VI) are represented (red lines). *VM* vastus medialis, *VL* vastus lateralis

Muscle thickness, the distance between two fasciae, is easily identifiable with ultrasound (Fig. 3). Its reliability has been previously reported in comparison with other imaging modalities as well as direct measurements on dissected cadavers [25, 47, 48], while its reproducibility has been defined as the highest in various muscles [26, 49, 50]. Since CSA is directly related to the loss of strength, some authors tried to predict CSA directly from muscle thickness. Although the two parameters significantly correlate [51], the prediction of CSA from muscles thickness has not been proven [26]. Thus, even if it has been shown that muscle loss of ICU patients could be monitored by thickness measurements [52], we suggest that other indexes reflecting muscle strength should be added to muscle thickness in order to improve precision.

### Echointensity

**Fig. 4:**
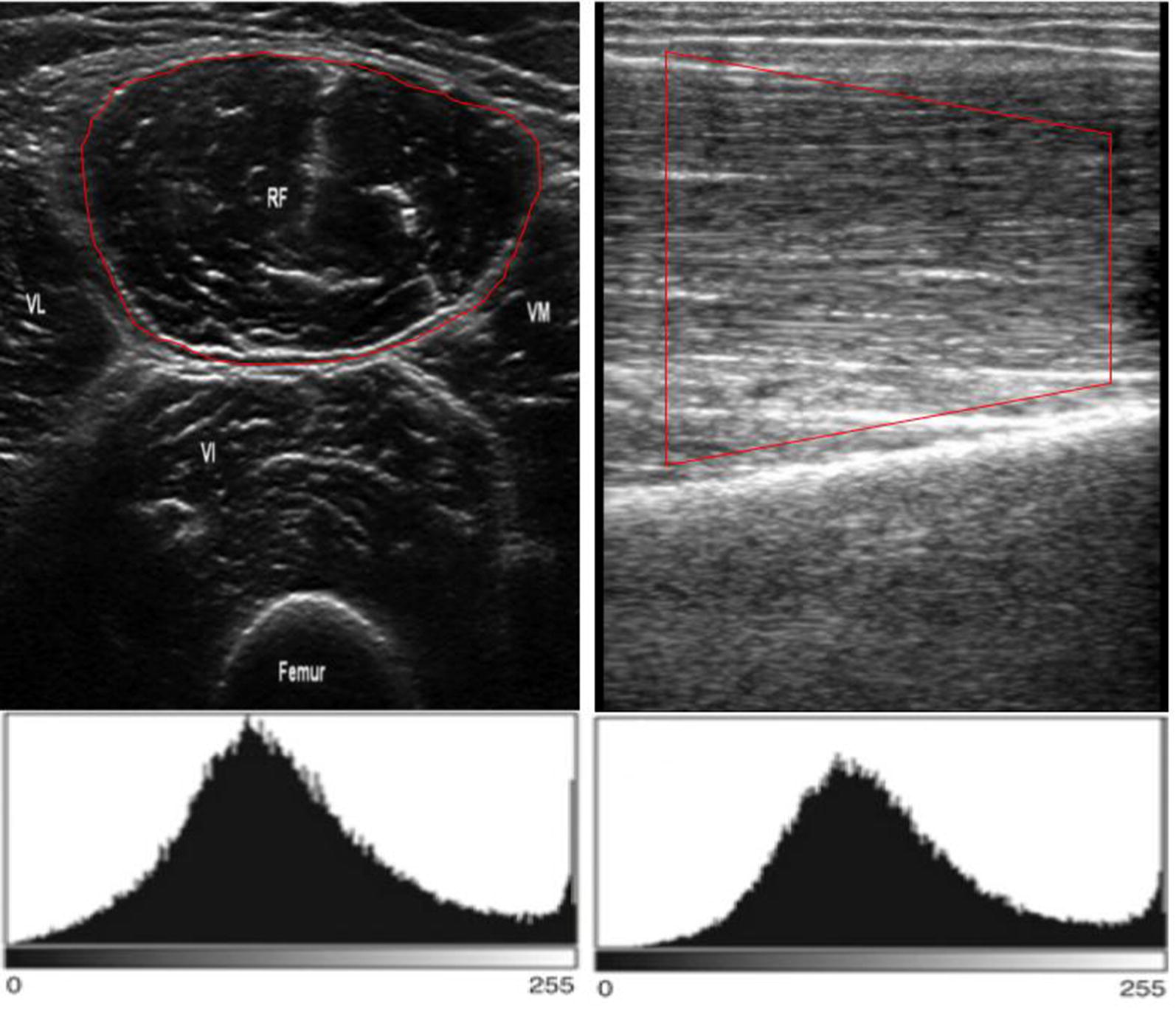
The muscle ultrasound echointensity. An example of the grayscale histogram in the transverse (right) and longitudinal (left) axis of the rectus femoris

Information about muscle composition can be gathered by quantification of muscle echogenicity [53]. The measure of the image grayscale reflect the muscle’s composition: increased echogenicity indicates more homogenous muscle [54]. Echointensity is calculated by performing grayscale analysis of image pixels. Briefly, all the pixels in a selected area of the muscle are categorized on a grayscale configuration using a standard histogram function widely available in many commercially available types of software for image editing (Fig. 4). Quantitative grayscale analysis has proven to be better than visual assessment alone of ultrasound images [37], but it is slightly more time consuming and requires the establishment of normal reference values. Ultrasonic echogenicity can be graded according to a score that classifies ultrasonic echogenicity semiquantitatively into four levels, with higher grades corresponding to increased severity of muscle impairment [55]. Graded echogenicity has been shown to correlate with muscle pathologic findings on biopsy [53]. As with other measures, echogenicity measurements are highly influenced by observer-dependent factors, such as the adjustment of the ultrasound probe. Additional, factors, such as hydration balance, might also have an impact.

### Pennation angle

**Fig. 5:**
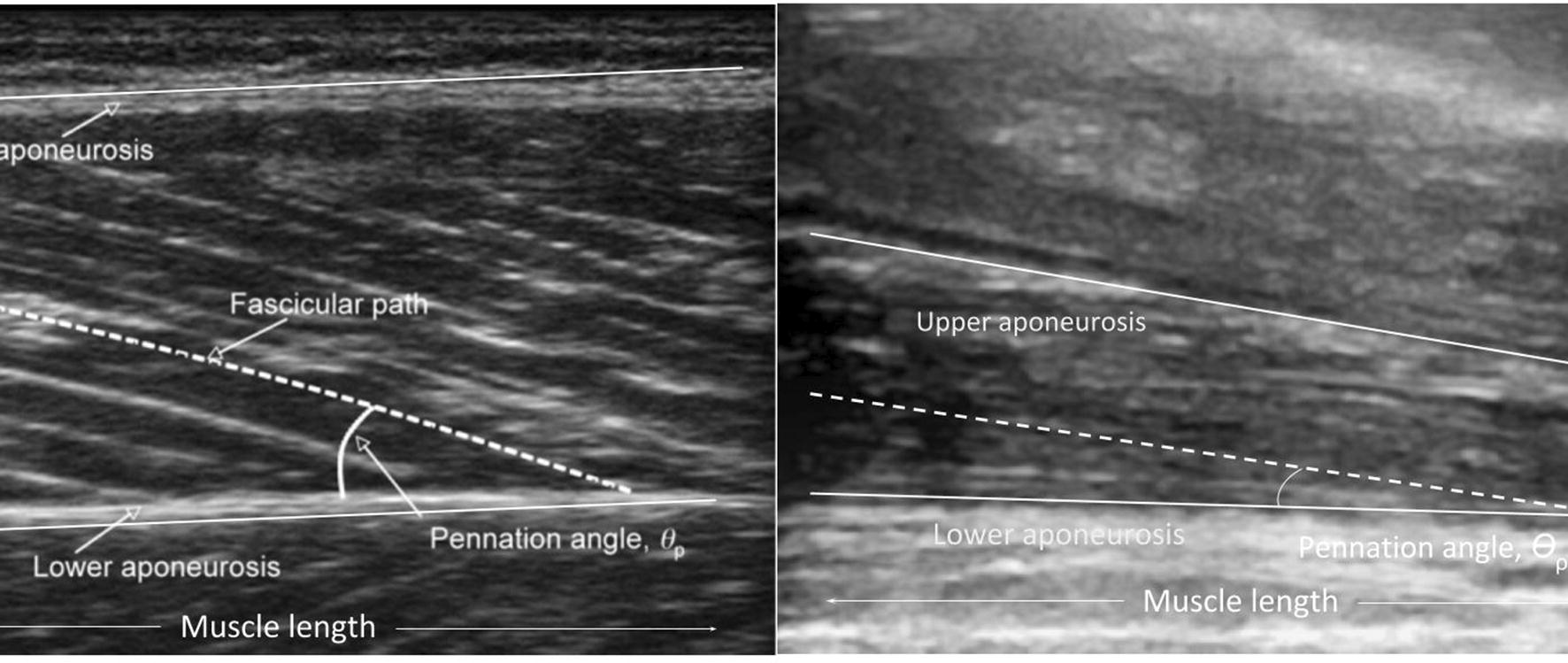
The muscle ultrasound pennation angle. The figure represents a longitudinal view of quadriceps rectus femoris muscle. The pennation angle is calculated between the intercept of fascicular path to the lower aponeurosis. Additionally, the muscle length can be measured. These two variables may be used to determine the strength of the muscle, as the lower is the angle, the lower is the length, and the lower is the strength. The right panel represents a representative reduction in pennation angle after 1 week of ICU stay

As mentioned above, muscle architecture can be described by the pennation angle, i.e., the angle of insertion of muscle fibers into the aponeurosis (Fig. 5). This angle provides information about muscle strength, as the greater the pennation angle, the more the contractile material packed within a given volume and by inference, the higher is the muscle’s capacity to generate force [56]. Moreover, pennation angle has been shown to be significantly correlated with the CSA [57]. Therefore, the angle of pennation is critical for determining force dynamics of muscle. Because pennation angle measurements are strongly influenced by adjustment of the ultrasound probe, some authors have expressed concerns regarding the observer dependency of this technique [58]. In particular, its reproducibility in muscles other than the quadriceps has been reported low [38]. Eventually, the fascicle length (FL) can be derived from pennation angle and muscle thickness, as described elsewhere [51] using the following formula: FL = TH/(sin PA), where TH is muscle thickness and PA the pennation angle. Muscles with larger pennation angles are thicker, as they have greater numbers of sarcomeres in parallel with the direction of the fascicle. It is possible that these parallel sarcomeres are lost first, causing loss of pennation angle as an indication of reduced thickness.

### Muscular ultrasound in clinical practice

Lower limbs muscles are more subject to early atrophy than those of the upper limbs [32]. The quadriceps, the largest muscle group of the lower limb, is the one generally explored with ultrasound. The image obtained allows assessments of muscle thickness, area, and ultrasound pattern, information, which can monitor contraction patterns that characterize muscular physiology and pathology. In the following section, we will describe the evidence on the use of this technique in critically ill patients (Table 1). Our comprehensive bibliographic search strategy accessed the following databases: PubMed, CINAHL, Cochrane Library, Scopus, Web of Science, from their inception to the cutoff date of July 31, 2018. The following keywords were used, alone or combined with appropriate Boolean operators, to search these databases: “muscular,” “peripheral muscular,” “ultrasound,” “intensive care unit,” “critical care,” “critical illness,” “weakness.”

**Table 1:** Principal studies regarding peripheral muscular ultrasound in the ICU setting

Study and year of publication	Design	Parameters	Main remarks
Reid 2004 [49]	50 ICU patients	Serial measurements of both mid-upper arm circumference (MAC) and muscle thickness, using ultrasound, were made at 1–3 day intervals	Muscle thickness decreased in almost every patients; ultrasound technique devised to identify muscle wasting in the presence of severe fluid retention works in the majority of patients; energy balance made no difference to the rate of wasting
Gruther 2008 [37]	118 ICU patients	Muscle layer thickness of the quadriceps femoris detected by US	Quadriceps femoris thickness showed a significant negative correlation with length of stay in ICU and seems to be higher during the first 2–3 weeks
Gerovasili 2009 [60]	49 ICU patients	Electrical muscle stimulation effects on cross-sectional diameter (CSD) of the vastus intermedius and the rectus femoris of the quadriceps muscle	The CSD of the right rectus femoris decreased significantly less in the EMS group, and the CSD of the right vastus intermedius decreased significantly less in the EMS group
Derde 2012 [61]	208 ICU patients	Markers of muscle atrophy and denervation versus rectus abdominis and vastus lateralis; tissue and electrical physiological analysis	Both limb and abdominal wall skeletal muscles of prolonged critically ill patients showed down-regulation of protein synthesis at the gene expression level as well as increased proteolysis
Puthucheary 2013 [15]	63 ICU patients	Serial US measurement of the rectus femoris cross-sectional area (CSA) on days 1, 3, 7 and 10; histopathological analysis was performed	There were significant reductions in the rectus femoris CSA observed at day 10
Cartwright 2013 [34]	16 ICU patients	Serial muscle ultrasound for thickness and grayscale assessment of the tibialis anterior, rectus femoris, abductor digit, biceps, and diaphragm muscles over 14 days	The tibialis anterior and rectus femoris had significant decreases in grayscale standard deviation when analyzed over 14 days. No muscles showed significant changes in thickness
Grimm 2013 [53]	28 ICU septic patients versus healthy	Biceps brachii and quadriceps femoris muscles, extensor muscles of the forearms and tibialis anterior muscle US, and nerve conduction studies on days 4 and 14 after sepsis	A significant difference in mean muscle echotexture between patients and controls was found at day 4 and day 14; day 4 to day 14, the mean grades of muscle echotexture increased in the patient group
Moisey 2013 [13]	149 ICU trauma patients	CT muscle cross-sectional area at the 3rd lumbar vertebra quantified and related to clinical parameters including ventilator-free days, ICU-free days, and mortality	Increased muscle index was significantly associated with decreased mortality
Baldwing 2014 [51]	16 ICU versus 16 healthy	Diaphragm, upper arm, forearm, and thigh muscle thicknesses US; respiratory muscle strength by means of maximal inspiratory pressure; isometric handgrip, elbow flexion, and knee extension forces with the use of portable dynamometry. Fat-free body mass (FFM) measured by bioelectrical impedance spectroscopy	Patients’ diaphragm thickness did not differ from that of the control group. Within the patient sample, all peripheral muscle groups were thinner compared with the diaphragm. Within the critically ill group, limb weakness was greater than the already significant respiratory muscle weakness
Puthucheary 2015 [62]	30 ICU patients	Vastus lateralis histological specimens and ultrasound assessment of rectus femoris echogenicity	Change in muscle echogenicity was greater in patients who developed muscle necrosis. The area under receiver operator curve for ultrasound echogenicity’s prediction of myofiber necrosis was 0.74. Myofiber necrosis and fascial inflammation can be detected noninvasively using ultrasound in the critically ill
Parry 2015 [54]	22 ICU patients	Sequential quadriceps US images were obtained over the first 10 days. Quadriceps muscle; CSA, TH, pennation angle and echointensity	There was a 30% reduction in vastus intermedius thickness, rectus femoris thickness, and cross-sectional area within 10 days of admission. Muscle echogenicity scores increased for both RF and VI. There was a strong association between function and VI thickness and echogenicity
Sarwal 2015 [44]	20 ICU patients	Diaphragm and quadriceps US muscle thickness and echogenicity	Excellent inter-observer reliability was obtained for all measurement techniques regardless of expertise level
Greening 2015 [56]	119 ICU COPD patients	Multivariate analysis between age, MRC dyspnea grade, home oxygen use, quadriceps (rectus femoris) cross-sectional area and hospitalization in the previous year	Patients with the smallest muscle spent more days in hospital than those with largest muscle. Smaller quadriceps muscle size, as measured by US in the acute care setting, is an independent risk factor for unscheduled readmission or death, which may have value both in clinical practice and for risk stratification
Mueller 2016 [55]	102 ICU postsurgical	Rectus femoris cross-sectional area US	Diagnosis of sarcopenia by ultrasound predicts adverse discharge disposition in SICU patients equally well as frailty
Turton 2016 [46]	22 ICU patients	Elbow flexor compartment, medial head of gastrocnemius and vastus lateralis muscle US at day 1,5 and 10th	No changes to the size of the elbow flexor compartment over 10 days. In the gastrocnemius, there were no significant changes to muscle. In the vastus lateralis, we found significant losses in muscle thickness
Segaran 2017 [58]	44 ICU patients	Muscle depth changes assessed by US on study days 1, 3, 5, 7, 12 and 14 in normal BMI versus higher	Obese patients lost muscle depth in a comparable manner to non-obese patients, suggesting that BMI may not prevent muscle depth loss
Annetta 2017 [57]	38 ICU trauma	Morphological changes of rectus femoris (RF) and anterior tibialis (AT) muscles up to 3 weeks	Progressive loss of muscle mass from day 0 to day 20, that was more relevant for the RF than for the AT; this was accompanied by an increase in echogenicity which is an indicator of myofibers depletion
Valla 2017 [64]	73 PICU	Transverse and longitudinal axis measurements of quadriceps femoris anterior thickness	Femoris thickness decrease, proposed as a surrogate for muscle mass, is an early, frequent, and intense phenomenon in PICU. Quadriceps femoris ultrasonography is a reliable technique to monitor this process and in future could help to guide rehabilitation and nutrition interventions
Hadda 2018 [60]	45 ICU patients	Arm muscle thickness US measured	There was an excellent intra- and inter-observer agreement among 5 observers for measurement of arm muscle thickness using bedside USG among patients with sepsis
Palakshappa 2018 [59]	29 ICU patients	RF CSA and TH versus muscle strength MRCs	RF CSA and TH decreased by 23.2% and 17.9% after 7 days. No correlation was found between US parameters and muscle strength test

### Muscular ultrasound in the ICU setting

Many studies examining the association between muscle weakness and clinical outcome have reported how muscle weakness was an independent predictor of mortality [59], increased ventilator-dependent time [60] and prolonged ICU length of stay (LOS) [61]. In particular, a negative correlation has been shown between muscular thickness (in both upper and lower limbs) and ICU LOS [52, 62, 63]. Several studies that investigated alterations in anabolic and catabolic signaling have introduced the use of ultrasound for the detection of muscular characteristics with the aims to improve pathophysiological knowledge of strength and to aid early diagnosis. However, interpretation of such reports is difficult because of significant methodological variability (such as small sample sizes and the lack of standardization of imaging assessment). Moreover, we are currently unaware of how sex, age and presenting illness affect loss of muscle mass in the critically ill. In most studies reliability of the ultrasonic technique was not the primary targeted outcome, which helps explain the lack of methodological consistency and precision. Some studies reported assessments of quantitative ultrasound reliability for measures of muscle linear depth, only two for CSA, and only one for echogenicity. Puthucheary et al. [16] showed a correlation coefficient (*R*^2^) of 0.97 for measuring rectus femoris CSA between two blinded independent raters. Baldwin published two observational methodological studies evaluating the reliability of muscle linear depth of mid-upper arm, mid-forearm, and mid-thigh and reported intra-rater intra-class correlation coefficients ranging from 0.998-1.0 [64] and ≥ 0.976 [65]. Regarding the muscle thickness, Gruther et al. [52] reported a coefficient of variation on repeated anterior thigh mean linear depth measures of 0.25%, without assessing the intra- or inter-rater reliability. More recently, Hadda [31] showed an excellent intra-observer (> 0.997) and inter-observer (0.963) agreement among five evaluators measuring arm muscle thickness. Finally, Grimm [66] also assessed muscle echogenicity, showing good inter-rater (0.915) and intra-rater (0.972) coefficients.

Another issue that should be taken into account is that most of the studies of ICUAW have been performed under the assumption that abnormalities found by the authors are ICU-acquired; no study attempted to control for prehospital muscle function or overall functional status as a predictor of ICUAW [67]. In fact, the so-called age-related frailty syndrome is characterized by a the loss of muscle mass that seems to be encountered in up to 80% of elderly ICU patients even in the absence of ICUAW diagnosis [68]. This condition constitutes an important predictor of long-term mortality [69] and morbidity [68], but the impact of pre-existing frailty on ICUAW is still unknown.

### Upper limb assessment

Most published studies investigated the lower limb muscles for the reasons explained above. Relatively few studies have selected the upper arm as their principal zone of interest, and when doing so sometimes compared it with other regions. Among these, Reid [66] performed serial measurements of mid-upper arm thickness within the first 72 h of ICU stay, showing how it decreased in almost every one of the 50 patients enrolled, independently of positive or negative energy balance. With a similar purpose, Baldwing et al. [65] more recently performed serial measurements of the thickness of the anterior mid-upper arm, mid-forearm in 16 septic ICU patients compared with healthy subjects. As expected, septic patients were significantly weaker than control participants, with significant differences recorded in the thickness and thickness/free fatty mass (FFM) of all peripheral muscles. Such data suggest that by 2 weeks of ICU admission, muscles of different functionality may not be equally affected by a combination of insults that occur during critical illness. Finally, Turton et al. [32] investigated the elbow flexor compartment, the medial head of gastrocnemius and the vastus lateralis muscle at admission and after 10 days in 22 ICU mechanically ventilated patients. Interestingly, this study showed no changes to the size of the elbow flexor compartment, and loss of muscle mass occurred preferentially in the lower limb. These data help justify interrogating the lower limb, as it appears to be the peripheral muscle group predisposed to develop early disuse atrophy among in critically ill patients. Moreover, the Turton study was the first to investigate the role of pennation angle. Patients who had a larger pennation angle at the day of admission had greater percentage reductions of pennation angle as well as of muscle thickness.

### Lower limb assessment

Focusing largely on lower limb ultrasound investigations, most published studies considered the muscle layer thickness and the CSA parameters, whereas only a few papers assessed ultrasonic muscle echogenicity as the key parameter of interest. In this regard, Grimm et al. [66] found significant alterations in muscle echostructure in the early stage of sepsis compared with healthy controls. Since those patients were septic and had a positive fluid balance, it is difficult to clarify to what extent the observed change in muscle echogenicity was caused by edema rather than muscle wasting. However, as the authors pointed out, the significance of tissue edema in the assessment of muscle echogenicity may be overestimated, since tissue edema cannot alter the bone signal that is part of the echogenicity score. Moreover, since the muscle echostructure score increased during the first 2 weeks of care despite a concomitant decrease in fluid balance, a specific structural damage in muscle architecture has been assumed. Cartwright and colleagues [70] found similar observations in echostructure changes over 2 weeks in both the tibialis anterior and rectus femoris muscles. Interestingly, these changes were similar to those seen in other myopathic conditions and included a significant increase in mean grayscale value, indicating an increased muscle echogenicity, and a decrease in grayscale standard deviation, indicating that the muscle became more homogeneous [71]. The use of grayscale standard deviation to define muscle homogeneity is justified, considering that the standard deviation decreases as the pixels in the region of interest become more uniform. However, once again, it is difficult to define whether this pattern of change occurred because of muscle breakdown and loss of the normally well-organized muscle architecture or due to inflammation or fluid retention in the subcutaneous tissue and muscle. Since it is not clear if the ultrasonographic muscle changes correlate with strength, Parry et al. [72] addressed this topic and reported that muscle echogenicity scores increased in quadriceps muscle (both rectus femoris and intermedious vastus) by 12% and 25%. Such observations suggest deterioration in muscle quality and establish a strong association between function and echogenicity. Eventually, in a recent prospective, two-center, observational study comparisons were made between sequential histological samples and ultrasound assessment of rectus femoris echogenicity [66]. This interesting paper showed how muscle echogenicity changes were greater in patients who developed muscle necrosis than in those who did not (8.2% vs. − 15.0%). In a previous study [16], rectus femoris CSA and protein/DNA ratio were assessed over time, suggesting that all decreased over the first week. Hence, lower limb muscle wasting has suggested to occur as a consequence of both depressed muscle protein synthesis and an elevation in protein breakdown relative to protein synthesis, resulting in a net catabolic state. Unfortunately, muscle ultrasound significantly underestimated protein loss (as measured by the protein/DNA ratio), perhaps in part because of the presence of interstitial edema. Moving forward on CSA studies, there is only one paper that integrated the ultrasound values into a sarcopenia and frailty prediction model, showing how the rectus femoris CSA, adjusted for sex and integrated with nutrition, comorbidities, depression, and patient demographics data, was able to predict adverse discharge disposition in surgical ICU patients [33]. With similar purpose, looking at the risk of unscheduled readmission or death, Greening et al. [73] demonstrated how smaller quadriceps muscle size described by CSA in the acute care setting was an independent risk factor for subsequent unscheduled readmission. CSA has been also evaluated in selected critically ill populations—such as trauma and obesity—confirming the previous observations. In particular, a 3-week follow-up analysis of CSA and muscle diameter followed in ICU trauma patients showed how 100% of them experienced severe muscle mass loss. Approximately 45% of rectus femoris muscle mass was lost by day 20, together with a progressive increase in echogenicity score [34]. The muscle depth as a measure of muscle wasting was compared among obese, overweight and normal-weight patients using a muscle ultrasound technique [74]. Compared with a previous study that used a similar methodology, the muscle depth loss was comparable and not statistically different between the groups at each of the interrogated time points. Lastly, muscle thickness of different muscle groups was investigated in many studies, and the main results in the majority indicated that it was significantly reduced. Among these, as already mentioned, a 0.2–5.7% decrease/day has been described for the upper arm [66], and a similar percentage in the lower limb [36]. Interestingly, the progression of this reduction was not uniform among the different quadriceps muscles, with a 30% reduction in rectus femoris and vastus intermedius thickness and 14% reduction in vastus lateralis [56]. Palakshappa [35] described the relationship between rectus femoris CSA and quadriceps muscle thickness, with volitional measures of strength and function at 7 days after the admission in ICU in 29 patients with sepsis. The authors observed an expected decrease in both rectus femoris CSA and thickness (23.2% and 17.9%, respectively) but established only a moderate correlation with strength on day 7. Similarly, Puthucheary showed that thickness measurements significantly underestimate ICU muscle wasting compared with rectus femoris CSA [75].

### Practical issue

**Fig. 6:**
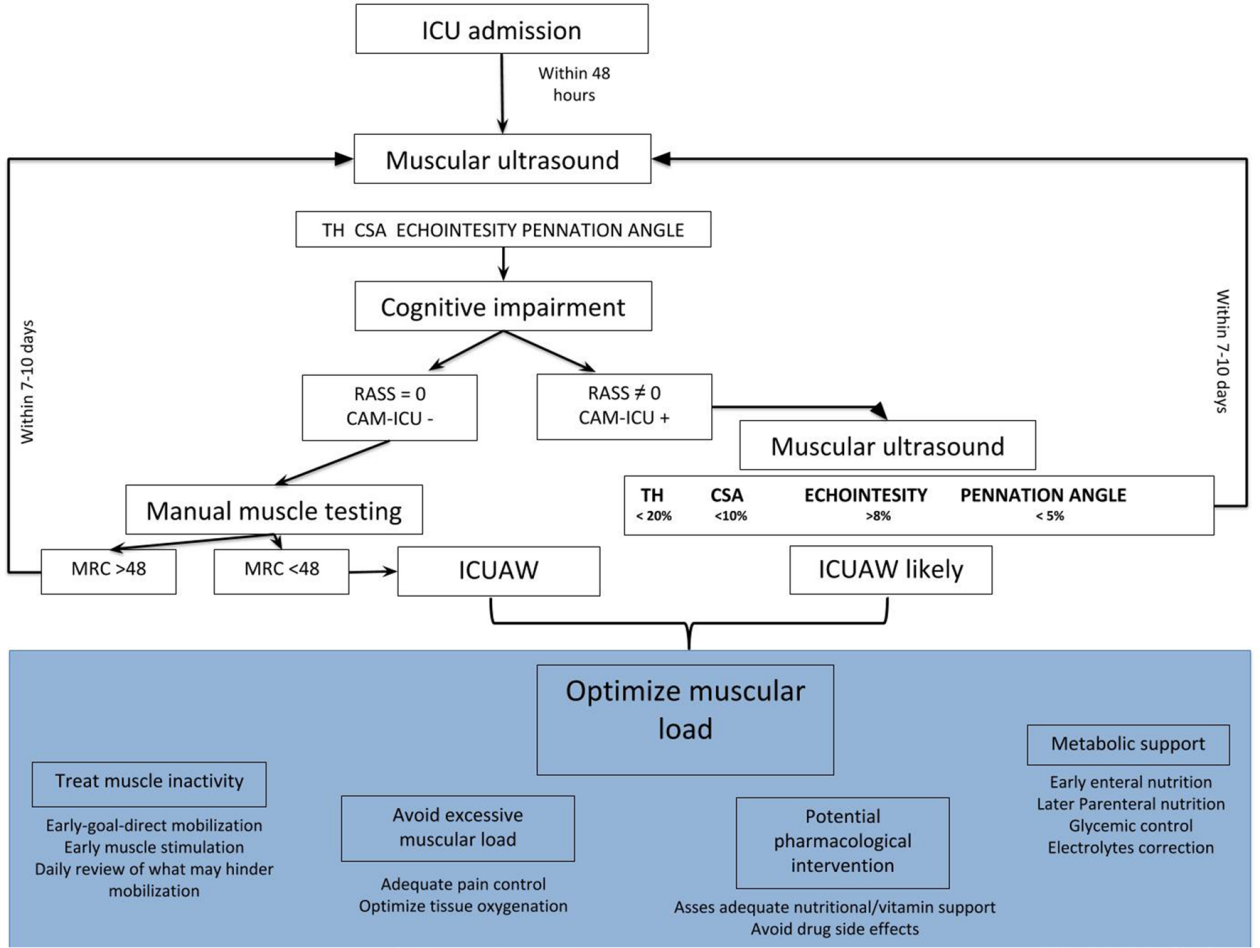
The muscle ultrasound flowchart for the assessment and minimization of ICUAW. This flowchart suggests a protocol for logical and early identification of ICUAW. Ideally, within the first 48 h, a first muscle ultrasound assessment should be performed for a baseline picture of patient muscle characteristics (the evaluation should at least regard the quadriceps rectus femoris, and it may be “omni-comprehensive” of muscle thickness (TH), cross-sectional area (CSA), echointensity (if the operator is familiar with any image editing software), pennation angle. At the same time, the cognitive impairment should be evaluated using standard reproducible scales (such as the Richmond agitation sedation scale and the confusion assessment method for the ICU). If these scores are in the normal range, the application of manual muscle testing such as the medical research council scale is possible. These first evaluations might be reconsidered within the first 7–10 days after the admission in the ICU, and their modifications over time, integrated with each other as well as with the reevaluation of MRC scale, allow an accurate diagnosis of ICUAW and should be used to modify the different patient-dependent factors, such as pharmacological strategies, muscular overloading or inactivity, and metabolic derangements. *RASS* Richmond agitation sedation scale, *CAM-ICU* confusion assessment method for the ICU, *ICU* intensive care unit, *MRC* Medical Research Council scale, *TH* muscle thickness, *CSA* cross-sectional area, *ICUAW* ICU-acquired weakness

Based on the current knowledge on this topic, we developed a methodological flowchart with the aim to diagnose ICUAW at an early stage and to optimize different patient-dependent factors, such as pharmacological strategies, muscular overloading or inactivity, and metabolic derangements (Fig. 6). Ideally, within the first 48 h after the admission to ICU, we suggest that a first muscular ultrasound assessment should be performed to paint a “baseline picture.” We also suggest confining the evaluation to the quadriceps muscle, and in particular to the rectus femoris. At the same time, volitional strength evaluation, using validated tools such as the Medical Research Council (MRC) scale [27, 43], should be performed as soon as cognitive impairment allows. The degree of possible cooperation should also be evaluated with validated scales for sedation, agitation level and delirium, such as the Richmond agitation sedation scale (RASS) and the confusion assessment method for the ICU (CAM-ICU) [76, 77]. In patients able to follow commands, manual muscle testing should be performed, with a score in the normal range confirming the absence of ICUAW. However, the necessary level of cooperation can reasonably be achieved on average only 8–10 days after ICU admission [72]. Impaired mental status or a low MRC score dictates the need for additional examination for ICUAW, with the aim of optimizing muscle load; in this case, serial reevaluations by muscular ultrasound may represent valuable tools. In this regard, reductions of 20% in muscle thickness, 10% of CSA, 5% of pennation angle and an increment in echointensity of at least 8% [66] seem reasonable indicators of ICUAW, even if the latter technique has not been standardized and no clear cutoff has yet been determined [72].

## Conclusions

Skeletal muscle wasting in the critically ill has significant functional implications for patients who survive, and the development of prophylactic or therapeutic interventions has been troubled by our lack of understanding of the pathophysiology driving the process of muscle wasting. Several studies have demonstrated that muscle ultrasound is able to reliably detect pathological changes, especially once it is performed repeatedly. Muscle ultrasound might help to identify those patients at highest risk of prolonged complications, which result from excess muscle catabolism. Despite this intriguing potential, the interpretation of the available studies is difficult because of significant methodological defects, inadequate sample sizes, and lack of standardization of the ultrasound methodology. Nevertheless, further studies are certainly needed to describe the detailed time course of ultrasonic muscle changes and the progression of spontaneous activity, particularly in relation to the functional clinical outcome.

## Data Availability

Not applicable.

## Abbreviations

ICU: intensive care unit
NMBAs: neuromuscular blockade agents
ICUAW: intensive care unit-acquired weakness
CT: computed tomography
MRI: magnetic resonance imaging
DEXA: dual-energy X-ray absorptiometry
CSA: cross-sectional area
TH: muscles layer thickness
FL: fascicle length
LOS: length of stay
FFM: free fatty mass
RF: rectus femoris
MRCs: Medical Research Council scale
RASS: Richmond agitation sedation scale
CAM: confusion assessment method
